# Mulholland Deformity With Pump Bump

**DOI:** 10.7759/cureus.12449

**Published:** 2021-01-03

**Authors:** Kishore Vellingiri, Nagakumar J S

**Affiliations:** 1 Orthopaedics, Sri Devaraj Urs Academy of Higher Education and Research, Kolar, IND

**Keywords:** mulholland deformity

## Abstract

Mulholland/Haglund's deformity is a symptomatic posterosuperior deformity of the heel. It affects middle-aged adults in general. It is more common in females than in males. It is mainly bilateral. The etiology is still poorly understood. The potential triggers may be a tight Achilles tendon, a high arch foot, and an inherited one. After the failure of conservative means, we record a case of bilateral Mulholland deformity showing dramatic improvement in pain following surgery.

## Introduction

Different names identify Mulholland deformity, such as retrocalcaneal exostosis, Haglund's deformity, and pump bump. It is marked by pain in the back of the foot that, after rest, is more apparent. For the most part, clinical examination and lateral ankle radiographs are appropriate for the diagnosis of Mulholland deformity [1]. After the failure of conservative means, we record a case of bilateral Mulholland deformity showing dramatic improvement in pain following surgery.

This case study was presented as a poster presentation at the 49th Annual Conference of the Indian Orthopaedic Association of Andhra Pradesh Orthopaedic Surgeons Society (OSSAPCON 2020) held in Vijayawada, India on January 31^st^ - February 2^nd^, 2020. The abstract of this article is published in the conference journal’s online supplement.

## Case presentation

**Figure 1:**
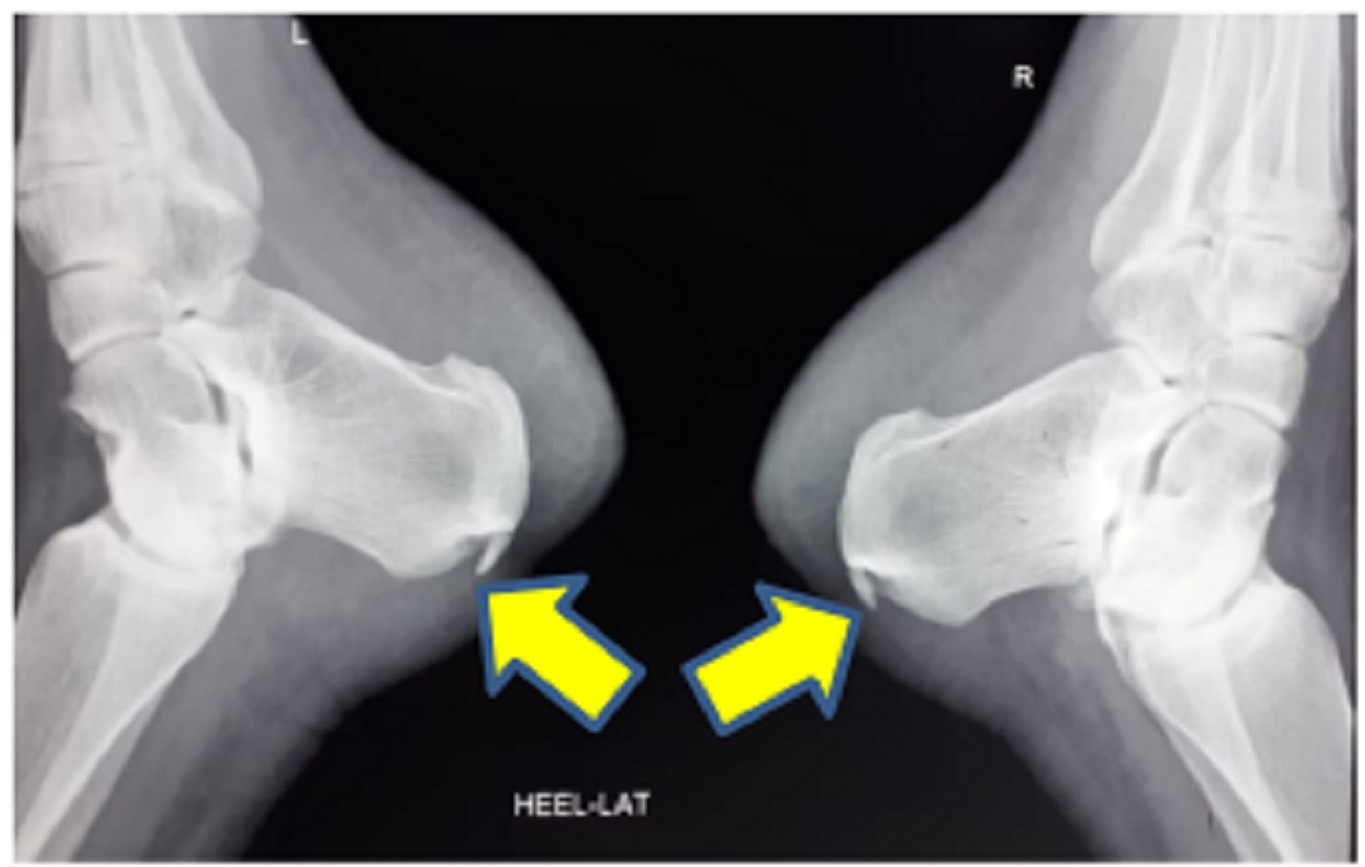
Lateral ankle radiograph showing bilateral Mulholland deformity

A 52-year-old male patient presented at our tertiary care hospital, in Kolar, India, with pain and swelling over the heel, pain during rest, and more during ambulation. Mulholland deformity/Haglund's deformity was diagnosed clinically and radiologically on examination. The patient’s lateral ankle radiograph is shown in Figure 1. The patient was initially treated conservatively for six months using anti-inflammatory drugs, analgesic agents, physiotherapy, and soft sole heels. The conservative medication did not relieve the pain. Patient underwent surgical operation via the removal of the calcaneal spur following informed and written consent. A lateral technique was used. An incision of 5 cm was made. The insertion of Achilles tendon found was resected along the lateral border revealing the prominent calcar tuber. The spur was removed using the osteotome and the edges smoothed using rongeur and rasp. The post-surgical phase was uneventful.

**Figure 2:**
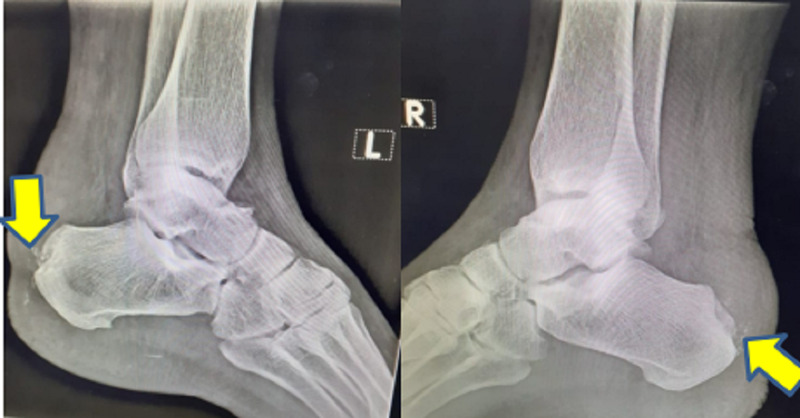
Postoperative lateral ankle radiograph following surgical excision

In terms of hospital treatment, the patient required intravenous amoxicillin-clavulanate twice daily for seven days, followed by oral amoxicillin-clavulanate potassium twice daily for seven days, depending on the patient’s post operative weight. Figure 2 demonstrates the postoperative lateral view radiography of the ankle joint. A short leg splint was put on the patient. For two weeks, nonweight bearing ambulation was prescribed to the patient and accompanied by suture removal. The surgical wound was healthy. With the walker, partial weight-bearing was initiated. And later on, weight-bearing began to be tolerated. The patient recovered from pain due to bilateral calcaneal spur/exostosis at final follow up, six months after surgery. The visual analog scale was eight. The discomfort was greatly diminished in the final follow up. Without any pain or discomfort, the patient would be able to do his everyday activities.

## Discussion

Mulholland deformity is a symptomatic posterosuperior deformity of the heel. It typically affects middle-aged adults in general. In females, it is more common than in males. It is mainly bilateral. The etiology is still poorly understood. The potential triggers may be a tight Achilles tendon, a high arch foot, and an inherited one [1]. Symptoms suggestive retrocalcaneal exostosis include pain localized to retrocalcaneal recess palpation only proximal to and anterior to the insertion of the Achilles tendon [2]. In addition to the Achilles tendon calcification and the osseous variations, the Fowler's angle and the parallel pitch lines were determined on a lateral view radiograph, such as a posterior calcaneal step spur or plantar osseous projections. For Haglund's syndrome, the Fowler's angle and parallel pitch lines were of no predictive benefit [3]. Reliable, objective diagnostic markers of bony deformity of the calcaneus and soft tissue affection in patients with posterior heel pain are parallel pitch lines, Chauveaux-Liet angle, ill-defined retrocalcaneal recess, superficial tendon Achilles bursa, and anteroposterior diameter of the Achilles tendon greater than 9 mm around 2 cm above insertion [4]. MRI does not affect the therapy of Haglund deformity patients. The resources of this cost-intensive and limited type of investigation can therefore be used elsewhere. An MRI can be useful in cases of atypical heel pain [5].

A local retrocalcaneal bursa corticosteroid injection can help with retrocalcaneal bursitis symptoms, but it poses a risk of rupture of the Achilles tendon. This risk-benefit must be considered when administering corticosteroid injections to professional and high-level athletes [6]. Posterosuperior calcaneal tubercle resection, bursectomy, Achilles tendon pathology excision, Achilles tendon reattachment, gastrocnemius aponeurotic recession, and flexor hallucis longus transfer were suggested treatment options for surgery [7]. For those who suffer from refractory Haglund's deformity, the lateral approach to calcaneal osteotomy may be an efficient procedure. The patient must, however, be made aware of the lengthy period of recovery [8]. Open and arthroscopic Haglund's osteotomy and dorsal close wedge calcaneal osteotomy may produce acceptable outcomes, but the latest development trend is minimally invasive treatment [9]. Latest techniques such as the endoscopic recession of gastrocnemius, radio-frequency Topaz repair of the coblation microtendon and repair of the Achilles suture, Bridge tendon are shown to increase the standard of reconstruction, shorten the postoperative recovery time and possibly increase the long term outcomes [10].

## Conclusions

Following surgical intervention for Mulholland deformity, the patient had better clinical and functional outcomes in our case study. Only after failed conservative management and patients with high demand activities such as running, surgical excision of the bony exostoses of the calcaneum is appropriate.
